# What do all the (human) micro-RNAs do?

**DOI:** 10.1186/1471-2164-15-976

**Published:** 2014-11-18

**Authors:** Alfred Ultsch, Jörn Lötsch

**Affiliations:** DataBionics Research Group, University of Marburg, Hans-Meerwein-Straße, 35032 Marburg, Germany; Institute of Clinical Pharmacology, Goethe - University, Theodor-Stern-Kai 7, 60590 Frankfurt am Main, Germany; Fraunhofer Institute of Molecular Biology and Applied Ecology - Project Group Translational Medicine and Pharmacology (IME-TMP), Theodor-Stern-Kai 7, 60590 Frankfurt am Main, Germany

**Keywords:** Micro-RNA, Gene expression, Regulation, Computational biology, Machine-learning, Knowledge-discovery, Genetics

## Abstract

**Background:**

Micro-RNAs (miRNA) are attributed to the systems biological role of a regulatory mechanism of the expression of protein coding genes. Research has identified miRNAs dysregulations in several but distinct pathophysiological processes, which hints at distinct systems-biology functions of miRNAs. The present analysis approached the role of miRNAs from a genomics perspective and assessed the biological roles of 2954 genes and 788 human miRNAs, which can be considered to interact, based on empirical evidence and computational predictions of miRNA versus gene interactions.

**Results:**

From a genomics perspective, the biological processes in which the genes that are influenced by miRNAs are involved comprise of six major topics comprising biological regulation, cellular metabolism, information processing, development, gene expression and tissue homeostasis. The usage of this knowledge as a guidance for further research is sketched for two genetically defined functional areas: cell death and gene expression. Results suggest that the latter points to a fundamental role of miRNAs consisting of hyper-regulation of gene expression, i.e., the control of the expression of such genes which control specifically the expression of genes.

**Conclusions:**

Laboratory research identified contributions of miRNA regulation to several distinct biological processes. The present analysis transferred this knowledge to a systems-biology level. A comprehensible and precise description of the biological processes in which the genes that are influenced by miRNAs are notably involved could be made. This knowledge can be employed to guide future research concerning the biological role of miRNA (dys-) regulations. The analysis also suggests that miRNAs especially control the expression of genes that control the expression of genes.

**Electronic supplementary material:**

The online version of this article (doi:10.1186/1471-2164-15-976) contains supplementary material, which is available to authorized users.

## Background

Micro ribonucleic acids (miRNAs), first described in 1993 [1], have been recognized as a major player in cellular regulation by conferring RNA interference [2]. MiRNAs are initially transcribed from host genes as longer primary transcripts or pri-miRNAs, from which shorter approximately 70 nucleotide-long pre-miRNAs are excised by the RNase III enzyme “Drosha” [3], pri-miRNA transcripts may code for more than one miRNA [4]. Pre-miRNAs are exported from the nucleus to the cytoplasm by the RNA-binding protein exportin 5 [5]. There, they are cleaved to the ~22 nucleotides-long mature miRNAs by the endoribonuclease “Dicer” [6]. Mature miRNAs impede gene translation by binding at complementary messenger RNA sequences, thereby initiating mRNA cleavage or obstructing mRNA incorporation in ribosomes.

More than 2000 human miRNAs have been identified [7, 8], potentially regulating the transcription of the 21,000 human protein-encoding genes [9]. Research during the last decade [10, 11] identified miRNAs dysregulations in several pathophysiological processes [12] such as cancer [13], cardiovascular diseases [14], viral infections [15] and pain [16]. In these and further context, miRNAs have been repeatedly found to modulate a wide range of physiological functions such as cellular differentiation, proliferation and apoptosis [17]. This suggests that miRNA-mediated control targets a range of typical biological processes hinting at a distinct systems-biology function of miRNAs.

The present analysis approached the role of human miRNAs from a genomics perspective and assessed the biological roles of those genes that can be considered to interact with miRNAs, based on empirically evidence [18, 19] or computational prediction [20]. Computational methods, publicly available databases and data mining tools (Table 1) were used to combine the knowledge about miRNA versus gene interactions with the acquired knowledge about higher-level organization of gene products into biological pathways [21], of which the gold-standard is the Gene Ontology (GO) knowledge base [22].

**Table 1:** **Publicly available data sources and freeware computational tools used to identify miRNA- targeted genes and to classify and visualize their biological functions (accessed November 22, 2013)**

Site name	URL	Reference
AmiGO (search utility for GO)	http://amigo.geneontology.org/	[23]
Gene Ontology (GO)	http://www.geneontology.org/	[22]
Gene Trail	http://genetrail.bioinf.uni-sb.de/	[24]
HUGO Gene Nomenclature Committee	http://www.genenames.org/	[25]
miRTarBase	http://mirtarbase.mbc.nctu.edu.tw/	[18]
NCBI gene index database	http://www.ncbi.nlm.nih.gov/gene/	
PubMed	http://www.ncbi.nlm.nih.gov/pubmed	
R software (version 3.0.2)	http://CRAN.R-project.org/	
TarBase database	http://diana.cslab.ece.ntua.gr/tarbase/	[19]
TargetScan Human	http://www.targetscan.org/	[7]

## Methods

### Empirical validated miRNA/gene interactions

**Figure 1:**
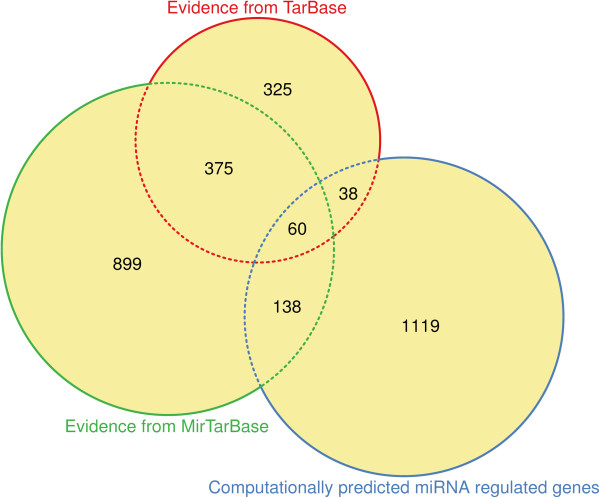
**Venn diagram** [26] **visualizing the sets of genes and the sizes of their intersections.** The present analysis was based on the miRNAs that resulted as the union of the three sources, i.e., evidence-based miRNA interacting genes from the miRTarBase database [18] evidence-based miRNA interacting genes from the TarBase database [19] and computationally predicted miRNA regulated genes based on an analysis using the TargetScan Human [20] software (for details of the prediction method, see appendix).

The genes likely to be regulated by miRNAs were identified by connecting several lines of evidence using publicly available computational methods, databases and data mining tools (Table 1). A first source of miRNA regulated genes consisted of empirically shown interactions of miRNA with genes. The majority of genes with empirical evidence for interaction with a miRNA was identified from miRTarBase database [18] that hosts the currently largest amount of experimentally validated miRNA versus target interactions. From this database the miRNA versus gene interactions were used for which strong experimental evidence was indicated, which in this database was defined as being provided in the form of reporter assays or western blots (file: miRTarBase_SE_WR.xls, Release 4.5 from http://mirtarbase.mbc.nctu.edu.tw/php/download.php). This gave a set of n = 360 different miRNAs acting on n = 1472 different genes. Additional miRNA regulated genes were queried from the TarBase database [19] that hosts further experimentally validated miRNA-gene interactions. In that database, experimentally validated, or supported, interactions are derived from specific, as well as high throughput experiments, such as microarrays and proteomics (for full details, see http://diana.cslab.ece.ntua.gr/?sec=home). From this database the reported direct interactions were used. This gave a set of n = 136 different miRNAs acting on n = 798 different genes. The size of unions and intersections of these gene sets are given in Figure 1.

### Computational prediction of miRNA/gene interactions

To reduce the impact of a possible research bias on the results, a second source of miRNA regulated genes was added from a computational prediction of miRNA regulated genes. A sufficiently credible prediction of miRNA regulated genes was obtained by querying the TargetScan Human software (version 6.2 [20]) for all human miRNAs known to this database. To obtain valid predictions an intensive correction against false positive predictions was performed. Considering the complexity of computational identification of miRNA targets [4, 27], a subsequent analysis of the distribution of the output of TargetScan, the so called ”Total Context^+^ scores” (TCP scores) [28, 29] was performed. To minimize the risk of false positive predictions, this distribution was compared with the scores for empirical validated miRNA targets and only those interactions were kept for which a probability of more than 98% for a valid interaction could be derived (Additional file 1). This filtering reduced the n = 14610 unique genes and n = 1539 human miRNA for which TargetScan predicted a miRNAa interaction to only n = 1355 genes and n = 548 human miRNA for which the computer prediction is sufficiently reliable. The union of the miRNA form empirical evidence and filtered computational predictions resulted in n = 788 different human miRNAs with interactions on n = 2954 different genes (Additional file 2: Table S1).

### Biological roles of miRNA regulated genes

To assess the role of miRNA regulation, the biological roles of the genes were identified based on the Gene Ontology (GO) knowledgebase [22] where the knowledge about genes is formulated using a controlled vocabulary of GO terms (categories), to which the genes [30] are annotated [31]. GO terms are related to each other by “is-a”, “part-of” and “regulates” relationships forming a polyhierarchy (i.e., a directed acyclic graph (DAG [32], knowledge representation graph). Particular biological processes, cellular localizations or molecular functions annotated to the miRNA-regulated genes were found by means of an over-representation analysis (ORA [33]) using the web-based GeneTrail [24] tool. This tool calculated the significance of the occurrence of the genes of the set of miRNA regulated genes at each term of the GO with respect to the expected occurrence of the genes given by all GO annotations. Statistical significance (p-values) was calculated by the GeneTrail program by applying Fisher’s exact test with Bonferroni α correction [34]. The result was a representation of the complete knowledge about the biological roles of miRNA-regulated genes (complete DAG). To perform this information more intelligible, functional abstraction [35] was applied identifying a special set of GO terms, i.e., “functional areas”, that represent the knowledge contained in the complete DAG at a maximum of coverage, certainty, information value and conciseness [35]. Finally, for GO terms describing biological processes the functional areas could be subsumed to topics to further enhance the conciseness of the description.

To assess the validity of the GO overrepresentation analysis (ORA) in a ten-fold repeated experiment n = 3000 genes were randomly chosen from the set of all n = 17794 genes for which GeneTrail contained annotations. For a p-value threshold of *t*_*p*_ = 0.05 and Bonferroni α correction none of these gene sets produced any significant go term. It could be observed that a small subset of miRNA interacts with many, i.e. up to 229, genes and on the other hand a large subset of miRNA (n = 304 of the n = 788 miRNA) interacts only with one gene. To address a potential bias of this unequal distribution the set of n = 788 miRNA was split into two separate subsets A and B. Set A contains 23% (n = 181) miRNA which interact with 75% of the n = 2954 genes. Set B contained the other miRNA that interacted with only a few (n <6) genes. Set A produced the same set of functional areas as the set of all n = 2954 genes with a median p-value of 1.0 · 10^−38^. Set B reproduced the functional areas of the set of all genes (median p-values of 1.0 · 10^-13)^ with the exception of “biological adhesion” and “response to stimulus” (details given in the supplement).

## Results

The analysis of the biological roles by the human miRNA regulated genes could be based on a total of 2954 genes obtained by unifying (Figure 1) the evidence-based sets of miRNA-interacting genes of n = 1472 queried from the miRTarBase database [18] and n = 898 queried from TarBase database [19]. This set of empirical evidences was augmented by n = 1355 genes obtained by computational prediction on the basis of the output of TargetScan [20] (Additional file 1). With overlaps between the gene sets (Figure 1), the analytical basis comprised of 62% evidence-based and 28% computationally predicted miRNA–regulated genes that based on the same sources of evidence or predictions interact with 344 different miRNAs.

This set of n = 2984 human genes empirically shown to be regulated by miRNAs or sufficiently credible computationally predicted to interact with miRNAs was used for an over-representation analysis (ORA [33]) with a p-value threshold of *t*_*p*_ = 1.0 · 10^−5^ and Bonferroni α correction. This resulted in a polyhierarchy of 187 significantly over-represented GO terms in GO categories “biological process”, “cellular component” and “molecular function” (Additional file 3: Figure S1, ORA_Empirical_plus_Predicted.png). By contrast, no under-represented terms were seen.

For the largest GO category, i.e., “biological process” containing 156 significant terms, functional abstraction [35] provided 17 functional areas (Table 2). This described the biological processes in which the genes that are influenced by miRNAs are involved by six major topics comprising biological regulation, cellular metabolism, information processing, development, gene expression and tissue homeostasis. The identified functional areas can be exploited to split the specific knowledge representation graph (DAG; Additional file 3: Figure S1, ORA_Empirical_plus_Predicted.png) of the 156 terms in the GO category biological process into smaller hierarchies (aspects). This is demonstrated below for the functional areas “cell death” (GO:0008219, Figure 2) and “gene expression” (GO:0010467, Figure 3).

**Table 2:** **Functional areas (GO terms of the category “biological process”), topically sorted (left column), of the genes interacting with miRNAs, i.e., for which a gene versus miRNA interaction has been experimentally shown, sorted for the number of genes included**

	Functional area	GO term ID	Number of genes			
			Fraction [%]	Observed	Expected	[− log10 p-val]
**BR**	Biological regulation	GO:0065007	38.9	1149	817	54.3
**Metabolism**	Primary metabolic process	GO:0044238	34.2	1009	805	20
	Cellular macromolecule metabolic process	GO:0044260	29.2	864	615	34.4
	Nitrogen compound metabolic process	GO:0006807	23.2	685	478	27.6
	Cellular biosynthetic process	GO:0044249	21	620	428	25.5
**Information transmission**	Signaling	GO:0023052	22.7	671	489	21
	Response to stimulus	GO:0050896	17	501	381	10.4
	Cell communication	GO:0007154	10.8	319	207	15.2
**Development**	Developmental process	GO:0032502	17.3	512	352	20.5
	Cellular component organization	GO:0016043	15.5	459	341	10.9
	Multicellular organismal development	GO:0007275	15.2	448	305	18
	Cell proliferation	GO:0008283	8.9	262	152	19.2
	Cellular component movement	GO:0006928	4.9	142	77	11.8
	Biological adhesion	GO:0022610	4.2	123	77	5.4
**GE**	Gene expression	GO:0010467	18.8	554	380	22.9
**TH**	Cell death	GO:0008219	8.2	243	138	18.8
	Cell cycle	GO:0007049	6.1	180	115	8.1

BR: biological regulation, GE: gene expression, TH: tissue homeostasis.

Significant and remarkable gene ontology (GO) terms (for definition see the AmiGO search tool for GO [23]) resulted from over-representation analysis (ORA) of the 2945 genes with experimentally shown or computationally predicted miRNA interaction that were annotated to the GO category “biological process”.

**Figure 2:**
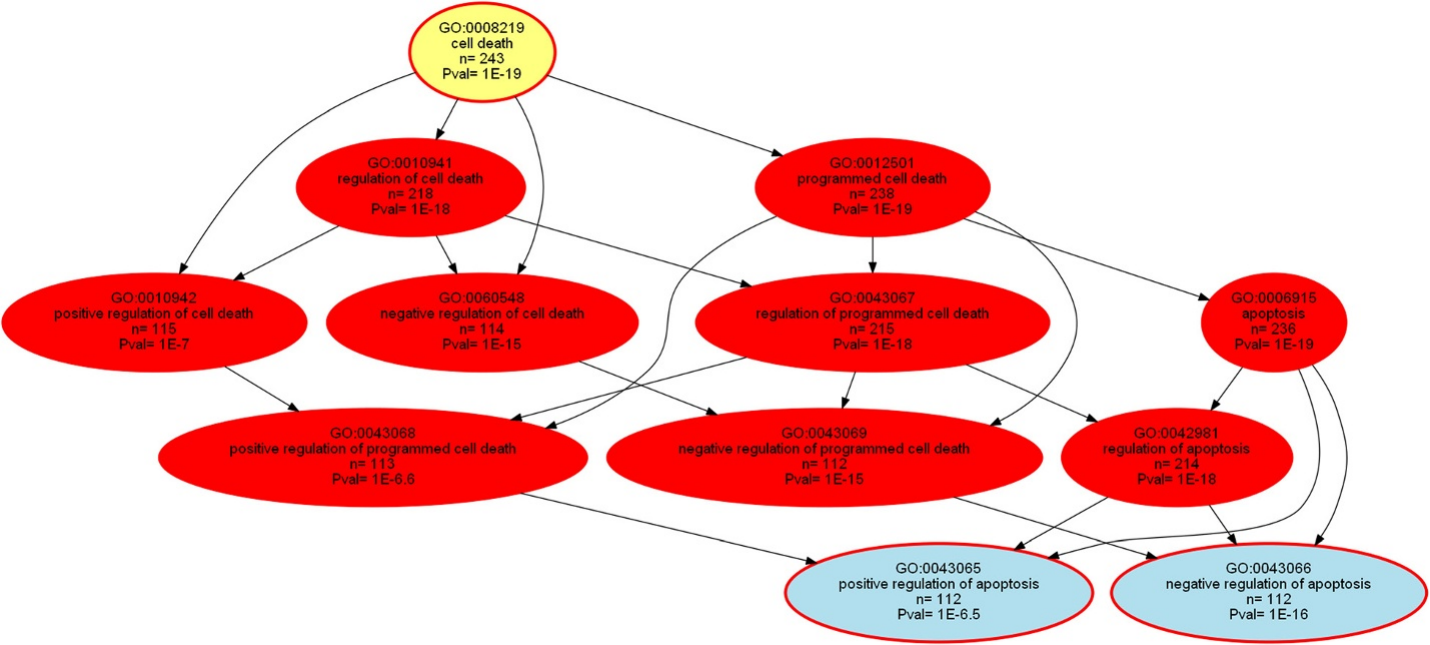
**Directed acyclic graph (DAG** [32]**) representing the nested Gene Ontology (GO) classification showing the polyhierarchy of functional annotations (GO terms) assigned in the GO category “biological process” to the 2954 genes (Figure** 1 **) that supported by empirical evidence from the miRTarBase** [18] **or TarBase** [19] **databases or computationally predicted using the TargetScan Human** [20] **software interact with miRNAs.** The figure is based on the GeneTrail web-based analysis tool [24] and represents the results of an over-representation analysis with parameters p-value threshold, *t*_*p*_ = 1.0 10^−5^ and Bonferroni α correction. The figure shows a particular aspect of the polyhierarchy, namely the ORA for the n = 243 (8% of all miRNA regulated genes) that were annotated with the GO term “cell death”. Significant terms are shown as red colored or framed ellipses, with the number of member genes indicated in line three, the expected number of genes in line five and the significance of the deviation between the two numbers given as minus log10 p. The functional area (Table 2) is indicated in yellow, and the leaves of this polyhierarchy at the select p-value threshold are shown in blue indicating the most specific significant GO terms. The vertical succession reflects the height of the terms in the GO polyhierarchy.

**Figure 3:**
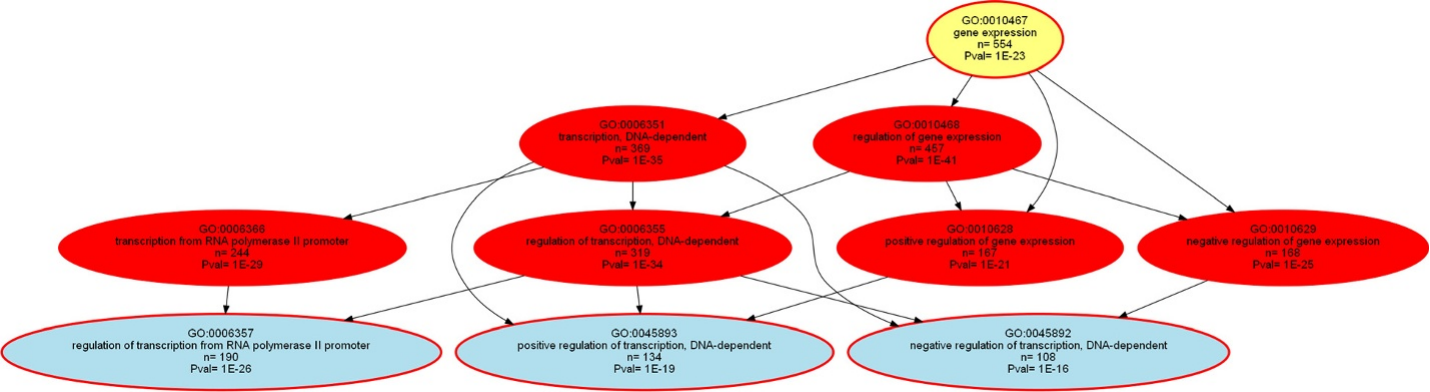
**Directed acyclic graph (DAG** [32]**) representing the nested Gene Ontology (GO) classification showing the polyhierarchy of functional annotations (GO terms) assigned in the GO category “biological process” to the 2954 genes (Figure** 1 **) that supported by empirical evidence from the miRTarBase** [18] **or TarBase** [19] **databases or computationally predicted using the TargetScan Human** [20] **software interact with miRNAs.** The figure is based on the GeneTrail web-based analysis tool [24] and represents the results of an over-representation analysis with parameters p-value threshold, *t*_*p*_ = 1.0 10^−5^ and Bonferroni α correction. The figure shows a particular aspect of the polyhierarchy, namely the ORA for the n = 554 (one fifth of all miRNA regulated genes) that were annotated with the GO term “gene expression”. Significant terms are shown as red colored or framed ellipses, with the number of member genes indicated in line three, the expected number of genes in line five and the significance of the deviation between the two numbers given as minus log10 p. The functional area (Table 2) is indicated in yellow, and the leaves of this polyhierarchy at the select p-value threshold are shown in blue indicating the most specific significant GO terms. The vertical succession reflects the height of the terms in the GO polyhierarchy.

**Figure 4:**
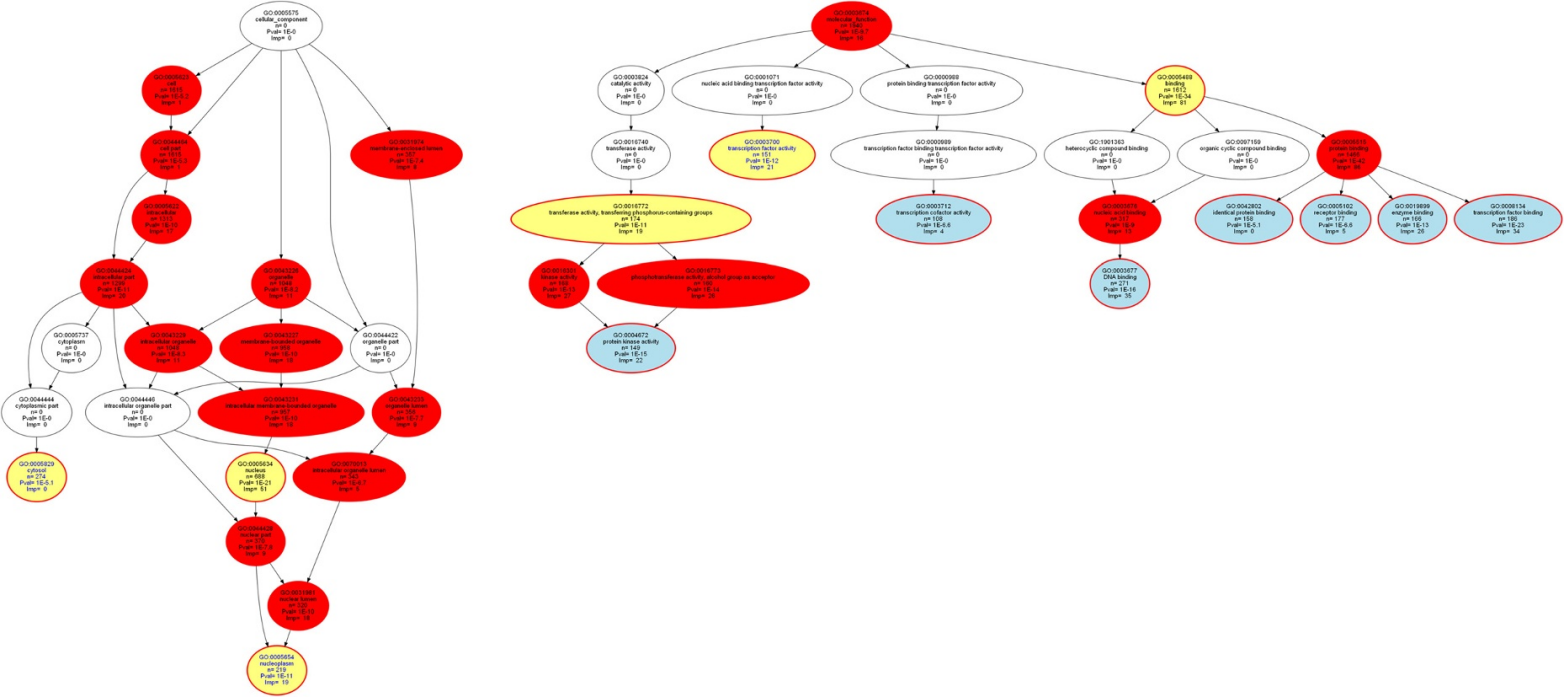
**Directed acyclic graphs (DAG** [32]**) representing the nested Gene Ontology (GO) classification showing the polyhierarchy of functional annotations (GO terms) assigned in the GO categories “cellular component” and “molecular function” (right) to the 2954 genes (Figure** 1 **) that supported by empirical evidence from the miRTarBase** [18] **or TarBase** [19] **databases or computationally predicted using the TargetScan Human** [20] **software interact with miRNAs.** The figure is based on the GeneTrail web-based analysis tool [24] and represents the results of an over-representation analysis with parameters p-value threshold, *t*_*p*_ = 1.0 10^−5^ and Bonferroni α correction. Significant terms are shown as red colored or framed ellipses, with the number of member genes indicated in line three, the expected number of genes in line five and the significance of the deviation between the two numbers given as minus log10 p. The GO category is indicated in yellow, and the leaves of this polyhierarchy at the select p-value threshold are shown in blue indicating the most specific significant GO terms.

Following functional abstractions of the further GO categories (Table 3) the GO category “cellular component” (Figure 4) indicated 2.5 times more miRNA-interacting genes annotated to the nucleus (n = 688 genes) than to the cytoplasm (n = 274). This significantly (p <10^−20^) exceeded the n = 504 genes that were expected to be annotated to the nucleus. Finally, the analysis of “molecular function” (Figure 4) indicated a particular role of miRNAs in selective, non-covalent interaction of a molecule with one or more specific sites on another molecule, i.e., “binding” (GO:0005488, p <10^−33^), including DNA binding (GO:0003677, p <10^−15^), and the regulation of “transcription factor activity” (GO:0003700, p <10^−10^) or “transcription factor binding” (GO:0008134, p <10^−23^).

**Table 3:** **Functional areas (GO terms of the categories “cellular component” and “molecular function”) of the genes interacting with miRNAs, i.e., for which a gene versus miRNA interaction has been experimentally shown, sorted for the number of genes included**

Functional area	GO term ID	Number of genes			
		Fraction [%]	Observed	Expected	[− log10 p-val]
**Cellular component**					
Nucleus	GO:0005634	23.4	688	505	20.8
Cytosol	GO:0005829	9.3	274	205	5.1
Nucleoplasm	GO:0005654	7.4	219	139	10.6
**Molecular function**					
Binding	GO:0005488	54.7	1612	1351	34.3
Transferase activity, transferring phosphorus-containing groups	GO:0016772	5.9	174	103	10.7
Transcription factor activity	GO:0003700	5.1	151	84	11.6

Significant and remarkable gene ontology (GO) terms (for definition see the AmiGO search tool for GO [23]) resulted from over-representation analysis (ORA) of the 2945 genes with experimentally shown or computationally predicted miRNA interaction.

## Discussion

Published literature attributes miRNAs to a systems biological role by a direct regulatory mechanism on classic protein coding genes, mainly by RNA interference impeding gene translation via destabilizing messenger RNA transcripts [36]. Considering that a miRNA may target many different genes and vice versa, a gene may be targeted by several different miRNAs [37, 38], the ~2000 miRNAs identified for *Homo sapiens*[7, 8] may potentially regulate the transcription of all 21,000 human protein-encoding genes [9] and thus be involved in any biological process known to the GO database. However, the present analysis suggested that only a seventh of the human genes seem to be miRNA regulated. Moreover, while the analysis also suggested that this regulation might be involved in any biological process, which is supported by the absence of under-represented GO terms in the ORA, the observed significant over-representation of GO terms clearly indicates that miRNAs play distinct biological roles, which exceed a general evenly-distributed function in gene regulation.

In the present work, a precise and comprehensive view of the systems biological role of miRNAs was obtained via analyzing the functions of a set of genes supported by published evidence for direct miRNA interaction [18, 19] combined with a trustworthy computational prediction of miRNA interactions. Using the knowledge about the biological processes, cellular localizations and molecular functions related to genes in the Gene Ontology (GO) knowledge base, the analysis provided a complete and precise description of the involvement of miRNAs in particular physiological and pathophysiological processes. The identification of these distinct roles, represented by functional areas (Tables 2 and 3), was a major finding of this analysis. These functional areas can be considered as a primary answer to the question “What do all those miRNAs do?” from a genomics point of view. Moreover, a further finding of this analysis was, that miRNAs, while exported from the nucleus as pre-miRNAs and in the cytosol processes to mature miRNAs where they exert their RNA interfering function, importantly regulate genes with products acting in the nucleus.

Several of the identified functional areas agree with the current knowledge about the involvement of miRNA in physiological and pathophysiological processes. Specifically, the topic “development” covers the roles of miRNAs reported in neuronal, muscle, and germline development, embryonic stem cell development and differentiation and immune development [17] in developmental regulatory pathways [39], neuronal specification and differentiation [40] or B cell development [41]. The topic information transmission containing the functional areas “signaling”, “response to stimulus” and “cell communication”, reflects the roles of miRNA regulation in immune response modulation and responses to immune-cell stimulation [17], response to stimuli [39, 42], autoimmune and inflammatory responses including the toll-like receptor pathway [40] and T cell receptor signaling [41].

The application of the comprehensive overview on the role of miRNAs in organisms may be demonstrated at two particular functional areas, namely cell death (GO:0008219) and gene expression (GO:0010467). The appearance of the first may be attributed to bias of present miRNA research, whereas the second leads to a possible new insight to the general purpose of miRNAs in organisms. MiRNAs are known to play a role in “cell death” including programmed cell death and apoptosis [17, 39, 40, 42, 43]. However, while the present analysis verifies this function, the suggestion that the regulation of cell death is a particular role of miRNAs, outstanding from their general role as a ubiquitous regulatory mechanism of gene expression, cannot be maintained when analyzing the evidence-based and computationally predicted sets separately. That is, cell death and related GO terms were only over-represented when analyzing the evidence-based gene set (Additional file 4: Figure S2, ORA_Empirical.png), suggesting a possible research bias since in the set of computationally predicted miRNA-interacting genes no GO term related to apoptosis emerged as significant (Additional file 5: Figure S3, ORA_Predicted.png).

By contrast, the functional area “gene expression” seems to be a particularly important biological role of miRNA regulation. This GO term was significant in both the evidence-based and computationally predicted gene sets (Additional file 4: Figures S2, ORA_Empirical.png, and 3, ORA_Predicted.png). About one fifth of the miRNA influenced genes (n = 554) are involved in the regulation of gene expression as reflected by the analysis of the GO category “biological process”. Moreover, the ORAs for the GO categories “cellular localization” and “molecular function” also seemed to converge to gene expression. That is, the molecular functions included an over-representation of transcriptional functions such as transcription factor regulation and DNA binding. The cellular components where the products of the miRNA regulated genes are located, were more often than expected found in the nucleus. When considering the definition of the GO term “gene expression” (GO:0010467) as the biological processes in which a gene's genomic sequence is converted into a mature gene product or products (proteins or RNA) from the production of an RNA transcript, the processing toward a mature RNA and the translation into proteins [23], miRNA-regulation covers it completely.

**Figure 5:**
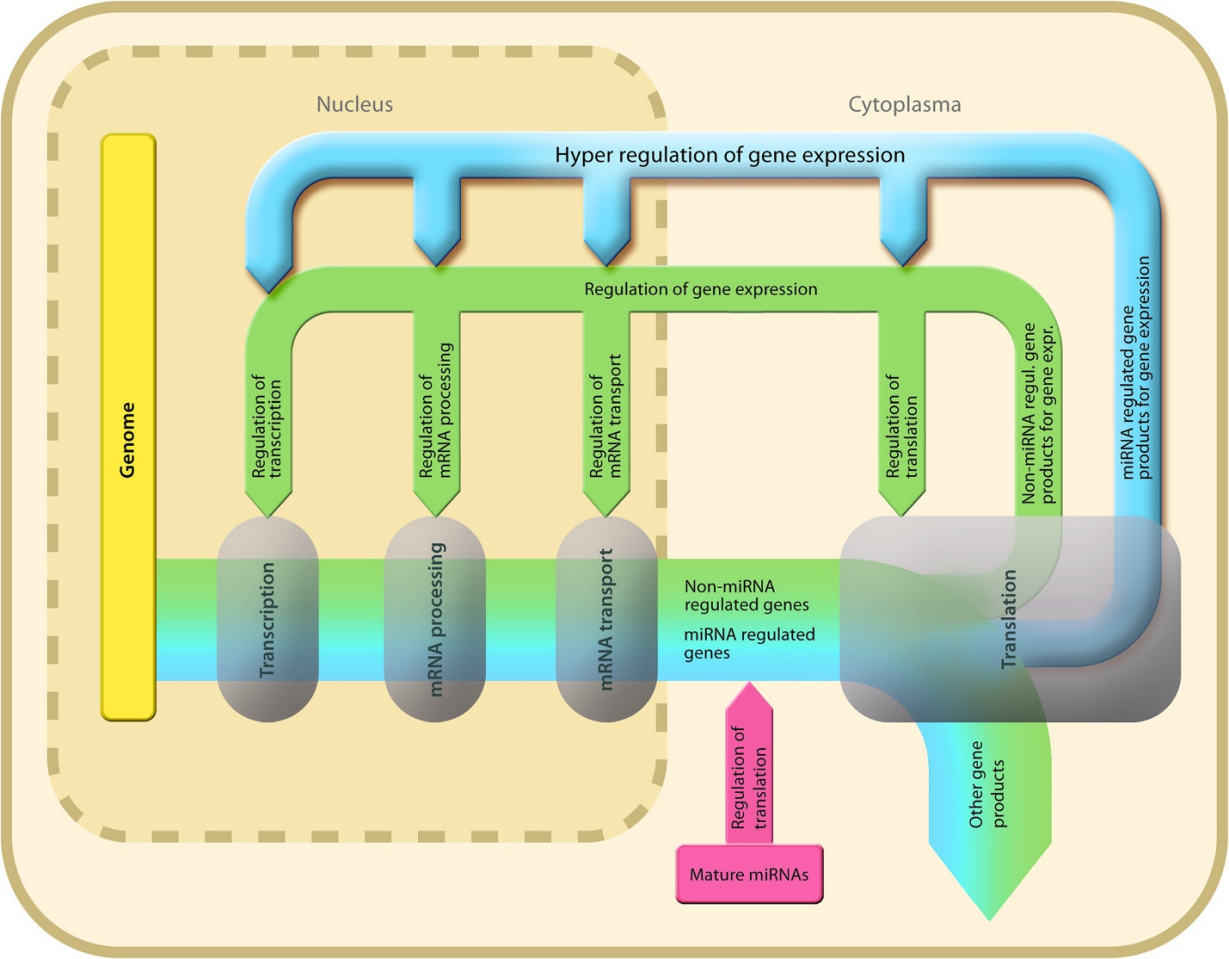
**Proposed “hyper-regulation” of gene expression by miRNAs.** The figure shows the role of miRNAs in the complex transcriptional network (blue arrow). By regulating (blue arrow) the expression of genes that are involved in the regulation of the expression of genes, a miRNA-dependent regulatory mechanism of gene regulation is formed on top of the miRNA-independent regulation of gene expression (green arrow). By this regulatory mechanism, proposed as “hyper-regulation” of gene expression (blue arrow), miRNAs interfere with the whole transcriptome mainly including intranuclear mechanisms besides the well-known extranuclear (red arrow) mechanisms. Hyper-regulation accommodates observations of global gene down-regulation in the absence of miRNAs [47] which downregulate gene product that reduce gene transcription such as DNA methyltransferases (Additional file 2: Table S1. RegulatedGenes_vs_miRNAs_Matrix.xlsx).

Thus, miRNA control applies in particular to the expression of genes that control the expression of genes, which we propose as “hyper-regulation” (Figure 5). The accepted role of miRNAs is the steering (inhibition) of the abundance of gene products, which is mechanistically exerting its functional infraction mainly in the cytoplasm. Hyper-regulation adds to mechanisms of gene expression control. It points at so far unappreciated increased complexity of gene expression control exceeding current paradigms. It can be hypothesized that miRNA mediated control represents an ancient major mechanism of cellular control providing small versatile molecules at comparably less metabolic effort for respective synthesis compared to protein translation. These systems are being found at all levels of gene expression from transcriptional fine-tuning. This was shown for the transcription activator Ets-1 where variable phosphorylation serves to fine-tune transcription at the level of DNA binding [44], the increasingly populated system of non-protein-coding regulatory RNAs increasing the diversity of control of genome dynamics and developmental programming [45], and the tight control of p53 as “guardian of the genome” shown to be closely regulated by miR-34a [46]. When considering that regulatory mechanisms may also repress genes that repress gene expression, such as all three DNA methyltransferases (DNMT 1, 3a and 3b; Additional file 2: Table S1. RegulatedGenes_vs_miRNAs_Matrix.xlsx), present findings also accommodate observations of genes being down-regulated following the deletion of dicer and thus abolishing the presence of miRNAs [47].

Based on the broad basis of current knowledge, the present data mining and computer science-based approach extends laboratory approaches to the role of miRNAs human biology. However, the analyses relied on external information and therefore, crucially depended on the accuracy and completeness of the empirical evidence entered into the queried databases. Limitation of possible research or publication bias was attempted by adding computational predicted miRNA/gene interactions (TargetScan), which were conservatively filtered to reduce false positives. The consequences of this have been discussed above, revealing that parts of the results cannot exclude a research bias whereas other parts such as the hyperregualtion of gene expression prevail regardless of the source of miRNA versus gene interactions. While the intention to exclude false positives nevertheless required conservative statistics throughout all analyses, the procedure might have triggered underestimations of the number of miRNAs versus gene interactions which could affect the results.

Finally, the present computational approach to the role of miRNAs emphasizes the increasing use of bioinformatics in the interpretation of miRNA functions. This accommodates the vast complexity of the acquired information about the role of miRNAs in biology and pathophysiology that probably exceeds human comprehension. Therefore, advances in research increasingly require computer science. This has been shown, for example, in two recent reports where current knowledge from databases was included in generating the research results via computational means. Specifically, the biological role of miRNAs found by array analyses in regenerating lungs was approached using integrative systems biology assessments including a GO analysis [48]. Interestingly, although this research was aimed towards the role of miRNAs in lung injury and tissue regeneration, one of the results was, that the GO term “gene expression” appeared as an important functional area of those genes that are influenced by the miRNAs particular identified in that experiments (see Figure six in [48]. Thus, the result that miRNAs seem to preferentially regulate genes that regulate the expression of genes obtained presently seems to appear in other analysis on a completely independent data basis as well, supporting its generality and improbability to merely present a bias in the presently queried evidence based miRNA versus gee interactions, which is further supported by the above-mentioned persistence of this results in the computationally predicted miRNAs regulated genes. A further recent example of the utility of computational biology is successful prediction of survival of glioblastoma patients by analyzing the inter-relation between miRNA and gene expression [49].

## Conclusions

Laboratory research identified contributions of miRNA regulation to several distinct biological processes. The present analysis transferred this knowledge to a systems-biology level. A comprehensible and precise description of the biological processes in which the genes influenced by miRNAs are notably involved was obtained. This identified seven different topics subsuming 17 functional areas for the genetic role of miRNA regulations: biological regulation, cellular metabolism, information processing, development, gene expression and tissue homeostasis. The present analysis explicitly intended to exploit all the current knowledge about miRNAs versus gene interactions and about the function of genes. This includes the knowledge gathered in databases and the computational means to make predictions. Indeed, the use of knowledge from different sources, when analyzed separately such as for the regulation of genes that regulate the expression of genes, agreed between empirical and predicted interactions, however, bears the potential of disagreements which need to be addressed in the laboratory. Therefore, the knowledge that has emerged from the present analysis can be employed to guide future research concerning the biological role of miRNA (dys-) regulations.

## Electronic supplementary material

Additional file 1: **An appendix with the detailed description of computational prediction of miRNA versus gene interactions.** (PDF 798 KB)

Additional file 2: Table S1: A cross-table displaying the miRNA versus gene interactions (RegulatedGenes_vs_miRNAs_Matrix.xlsx). (XLSX 102 KB)

Additional file 3: Figure S1: Displaying the results of overrepresentation analysis of the set of genes with experimentally shown or computationally predicted miRNA interaction: ORA_Empirical_plus_Predicted.png. (PNG 3 MB)

Additional file 4: Figure S2: Displaying the results of overrepresentation analysis of the set of genes with experimentally shown miRNA interaction: ORA_Empirical.png. (PNG 5 MB)

Additional file 5: Figure S3: Displaying the results of overrepresentation analysis of the set of genes with computationally predicted miRNA interaction: ORA_Predicted.png. (PNG 1 MB)
