# Supplementary material for: What do all the (human) micro-RNAs do?
Source: BMC Genomics. 2014 Nov 18;15(1):976. doi: 10.1186/1471-2164-15-976 (PMC4289375; doi:10.1186/1471-2164-15-976)

Supplementary materials

Computational prediction of miRNA targeted genes

The prediction of miRNA versus gene interactions was based on querying the TargetScan Human software (version 6.2 [1]). This program is one of the most used prediction programs for miRNA versus gene interactions [2]. The set of all human miRNA (n = 1,539) known to this program was used to predict miRNA versus gene interactions. The result of the calculation were numerical values, called "total context^{+} scores" (TCP scores) [3, 4], for n = 371,557 different miRNA versus gene interactions. The TCP scores combine the contributions of six aspects of miRNA versus gene interaction [4], i.e., site type, site number, site location, 3' pairing, local AU base pair content and seed-pairing stability. The obtained TCP scores ranged from -8.74 to 0 with the assertion that more negative values are associated with a bigger chance of a miRNA versus gene interaction as confirmed previously to a certain extent [2]. Only n = 288, i.e., less than 0.08 % of TCP scores are smaller than -1.

However, the acceptance of all nonzero TCP scores as assertion of a miRNA versus gene interaction would result in an unacceptably high rate of false-positive results. This problem was addressed by pursuing two different approaches: (i) comparison with empirically validated interactions and (ii) detailed analysis of the distribution of the TCP scores. All calculations were performed using the Matlab software (MathWorks, Natick, MS, USA). In detail, a query of the TarBase [5] and mirTarBase [6] databases resulted in n = 4,229 empirically validated miRNA versus gene interactions. To analyze the distributions, all TCP scores were rescaled to a positive range and zero invariant logarithmic transformed as "**L**ogarithmic **T**argetScan **S**cores" (LTS), with $\mathrm{LTS} = \log\big((-100 \cdot \mathrm{TCP}) + 1\big)$. The resulting distribution is shown as black line in Supplementary Figure 1 using the PDE kernel density estimation [8]. This distribution of TargetScan's LTS could be modelled by a Gaussian mixture model (GMM) with a mixture of three Gaussians, see the magenta lines in Supplementary Figure 1. The parameter of the model were optimized using the expectation maximization (EM) algorithm [7]. The values of the optimized model is given in Supplementary Table 1. Supplementary Figure 2 (left panel) shows the quality of the

GMM using a quantile/quantille (QQ-) plot. The Gaussians G1 and G2 with m = 1.2496 and m = 2.6005 represent a low or moderate probability that an interaction between a miRNA and a target gene occurs. Whereas the Gaussian G3 with m = 3.3807 represents a presumably intensive interaction between a miRNA and a target gene. Using the theorem of Bayes the probability p(miRNA interacts with gene) could be defined as the posteriori p(LTS is in G3) with respect to this GMM = (G1,G2,G3). This gives a lower bound of valid predictions of LTS > 3.0, see the vertical green line in Supplementary Figure 1. The validity of this lower bound is addressed by taking the empirical validated gene versus miRNA interactions into account. Using the same approach as described above the n = 4,229 empirical validated interaction could also be modelled by a GMM with three Gaussians. The parameters were again optimized using the using the expectation maximization (EM) algorithm [7] and the parameter values and model quality given in Supplementary Table 1 and Supplementary Figure 2, right panel.

As relatively few empirical validated interactions (n = 4,229) contrasted with many TargetScan predictions (n = 371,557), a Bayesian approach to derive a lower limit of the LTS to avoid false predictions would not deliver reliable results. However, a comparison of the models the distributions allowed addressing the validity of TargetScans predictions (Supplementary Figure 3). That is, the distribution of empirical validated interactions showed a large proportion of its mass at the high end of all TargetScan LTS values (green line in Supplementary Figure 3). This confirms that larger LTS values, i.e. larger negative "total context$^+$ scores", indeed correspond to a bigger likelihood of a miRNA versus gene interaction. This maximum likelihood approach allowed deriving the lower limit of LTS at a value of 3.06 from which the probability density function (PDF) of empirical validated interactions exceeds the PDF of all TargetScan scores.

The position of the Gaussian components of the GMM for all TargetScan predictions suggested the following interpretation: the first Gaussian $N(m_1=1.3, s_1, w_1)$ represented *LTS* (respectively *TCP*) values around zero for which no reasonable gene versus miRNA interaction. The second Gaussian

$N(m_2=2.6,s_2,w_2)$ described interactions of uncertain validity, while the third Gaussian $N(m_3=3.4,s_3,w_3)$ represented scores with a strong plausibility of indicating a gene versus miRNA interaction.

Using the theorem of Bayes, the prediction of a probability $p(g\#m|LTS(g,m)$ that miRNA m does not interact with a gene g based on the $LTS(m,g)$ score predicted by TargetScan was $p(m\#g|x) = p\big(m\ does\ not\ interact\ with\ g\big|LTS(m,g)\big) = 1 - posterior\big(LTS(m,g), \mathrm{N}(\mathrm{m}_3, s_3, \mathrm{w}_3)\big),$ where $posterior(x, N(m,s, w))$ denotes the Bayes posterior of the Gaussian component $N(m,s, w)$ with respect to the GMM. This probability represented a suitable p value of an interaction to be predicted by pure chance (Supplementary Figure 4). A limit of $p(m\#g|x) < 0.02$ leads to a threshold for acceptable predictions of $t_0 = 4.06$. To further minimize the rate of false positive results, a TargetScan prediction of a miRNA versus gene interaction for gene g was only accepted, if there were at least two different predictions with LTS > t_0. This approach resulted in a restrictive filtering of the output of TargetScan with the aim to avoid false positive predictions. It identified n = 6,805 acceptable predictions for miRNA/gene interactions. On the basis of this analysis, a set of n = 1,355 different genes was obtained for which it seems to be safe to assume that TargetScan predicts reliably a valid miRNA interaction.

Assessing the distribution of miRNA versus gene interactions

It could be observed that the number of genes which are the target of a miRNA are quite unequal. Only a few miRNA interact on many genes, while many miRNA target only one gene. It could be observed that a small subset of miRNA interacts with many, i.e., up to 229, genes and on the other hand a large subset of miRNA (n = 304 of the n = 788 miRNA) interacts only with one gene. To address a potential bias of this unequal distribution the set of n = 788 miRNA was split into two disjunct subsets A and B on the basis of a so-called ABC analysis [8]. An ABC curve is a plot of the faction of the largest number of MIRNA per gene interaction versus the fraction of the number of miRNA per genes (see Supplementary Figure 5). The sets were determined by the minimum distance from the ABC curve to the point of strongest inequality (0,1). This resulted in set A containing n = 181 miRNA (i.e. 23 %) which interacted with a total of n = 2,227, i.e. 75 %, of which n = 2,169 are unique; gene set A) genes. The remaining 77

% of miRNA were included in set B. These miRNA interact with n = 1,076 different genes (gene set B). For both gene sets A and B an ORA was calculated using a p-value threshold of $t_p = 1.0 \cdot 10^{-3}$ and Bonferroni α correction. The functional abstraction on the ORA for gene set A reproduced all functional areas that were present in all the union of gene sets A and B (AB) with a maximum p-value of $t_p = 1.0 \cdot 10^{-16}$ and a median p-value of $t_p = 1.0 \cdot 10^{-16}$. The ORA for gene set A reproduced 15 of the 17 functional areas that were present in all the union of gene sets A and B with a maximum p-value of $t_p = 1.0 \cdot 10^{-4}$ and a median p-value of $t_p = 1.0 \cdot 10^{-13}$. The two functional areas "biological adhesion" and "response to stimulus" were not significant at the p-value threshold of $t_p = 1.0 \cdot 10^{-3}$ for gene set B.

A calculation of precision, i.e. the fraction of number of terms in the ORA for gene set A that are also in AB versus the number of terms in the ORA for gene set A, and recall, i.e. the fraction of number of terms in the ORA for gene set A that are also in AB versus the number of terms in the ORA for gene set AB, was performed for all possible values of the p-value threshold t_p [9].The values of recall and precision at the largest F-measure [9] gave a recall of 69 % and a precision of 95 % for gene set A. The same calculation for gene set B resulted in a recall of 77 % and a precision of 81 %. These measurements demonstrate that the results of the ORA of the gene subsets A and B coincide to a sufficiently large extend with the ORA of all miRNA influenced genes considered in this work.

References Supplement

1. Lewis BP, Burge CB, Bartel DP: **Conserved seed pairing, often flanked by adenosines, indicates that thousands of human genes are microRNA targets**. *Cell* 2005, **120**(1):15-20.

2. Witkos TM, Koscianska E, Krzyzosiak WJ: **Practical Aspects of microRNA Target Prediction**. *Current molecular medicine* 2011, **11**(2):93-109.

3. Grimson A, Farh KK, Johnston WK, Garrett-Engele P, Lim LP, Bartel DP: **MicroRNA targeting specificity in mammals: determinants beyond seed pairing**. *Molecular cell* 2007, **27**(1):91-105.

4. Garcia DM, Baek D, Shin C, Bell GW, Grimson A, Bartel DP: **Weak seed-pairing stability and high target-site abundance decrease the proficiency of lsy-6 and other microRNAs**. *Nat Struct Mol Biol* 2011, **18**(10):1139-1146.

5. Papadopoulos GL, Reczko M, Simossis VA, Sethupathy P, Hatzigeorgiou AG: **The database of experimentally supported targets: a functional update of TarBase**. *Nucleic Acids Res* 2009, **37**(Database issue):D155-158.

6. Hsu S-D, Tseng Y-T, Shrestha S, Lin Y-L, Khaleel A, Chou C-H, Chu C-F, Huang H-Y, Lin C-M, Ho S-Y *et al*: **miRTarBase update 2014: an information resource for experimentally validated miRNA-target interactions**. *Nucleic Acids Res* 2014, **42**(Database issue):D78-85.

7. Dempster AP, Laird NM, Rubin DB: **Maximum Likelihood from Incomplete Data via the EM Algorithm**. *Journal of the Royal Statistical Society Series B* 1977, **39**(1):1-38.

8. Dickie HF: **ABC Inventory Analysis Shoots for Dollars, not Pennies**. *Factory Management and Maintenance* 1951, **6**:92-94.

9. Powers DMW: **Evaluation: From Precision, Recall and F-Factor to ROC, Informedness, Markedness & Cor-relation**. *J Machine Learn Tech* 2007, **2**(1):37-63.

10. Ultsch A: **Pareto Density Estimation: A Density Estimation for Knowledge Discovery.** In: *Innovations in Classification, Data Science, and Information Systems - Proceedings 27th Annual Conference of the German Classification Society (GfKL): 2003; Berlin*. Springer.

Supplementary Table 1: Parameter values of the Gaussian mixture model (GMM) for all rescaled and logarithmic TargetScan [1] (LTS) scores. The GMM is given as $p(x) = \sum_{i=1}^{M} w_i \cdot \frac{1}{\sqrt{2\pi s_i}} \cdot e^{-\frac{(x-m_i)^2}{2s_i^2}}$; $\sum w_i = 1$, with a number of mixes $M = 3$, where m_i, s_i and w_i are the parameters mean, standard deviation and relative weight of each of the Gaussians, respectively. The first Gaussian $N(m_1,s_1)$ represented LTS values around zero, i.e., no reasonable gene versus miRNA interaction. The second Gaussian $N(m_2,s_2)$ described interactions of uncertain validity, and the third Gaussian $N(m_3,s_3)$ represented scores with strong plausibility of indicating a gene/miRNA interactions. The parameters of the GMM were adapted to the date using the expectation maximization algorithm [7].

	Unacceptably weak scores	Uncertain validity	Interaction plausible
**GMM for All TargetScan LTS scores**			
m_i	1.2496	2.6005	3.3807
s_i	0.5984	0.4596	0.442
w_i	0.2252	0.3909	0.3839
**GMM for LTS scores of empirically validated interactions**			
m_i	1.2758	2.8686	3.573
s_i	0.4453	0.5431	0.3853
w_i	0.0671	0.4258	0.4816

Supplementary Figure 1: Distribution of logarithmic TargetScan [1] (LTS) scores, Gauss Mixture Model and Bayes Posterior, based on estimation of the Gaussian mixture model (GMM) LTS, given as p(x) = $\sum_{i=1}^{M} w_i \cdot \frac{1}{\sqrt{2\pi s_i}} \cdot e^{-\frac{(x-m_i)^2}{2s_i^2}}$; $\sum w_i = 1$, with a number of mixes M = 3, where m_i, s_i and w_i are the parameters mean, standard deviation and relative weight of each of the Gaussians, respectively. Demonstration of the description of density distribution of the TargetScan derived LTS (black line). The density has been described using the Pareto Density Estimation (PDE) as is a kernel density estimator particularly suited to discovery GMM [10]. The GMM is shown with the three Gaussians (magenta lines) that added up to the overall density estimate of LTS (blue line). To obtain the latter, i.e., highly likely scores indicating gene versus miRNA interaction, the Bayesian probabilities between the third with respect to the GMM were calculated (red line, see also Supplementary Table 1).

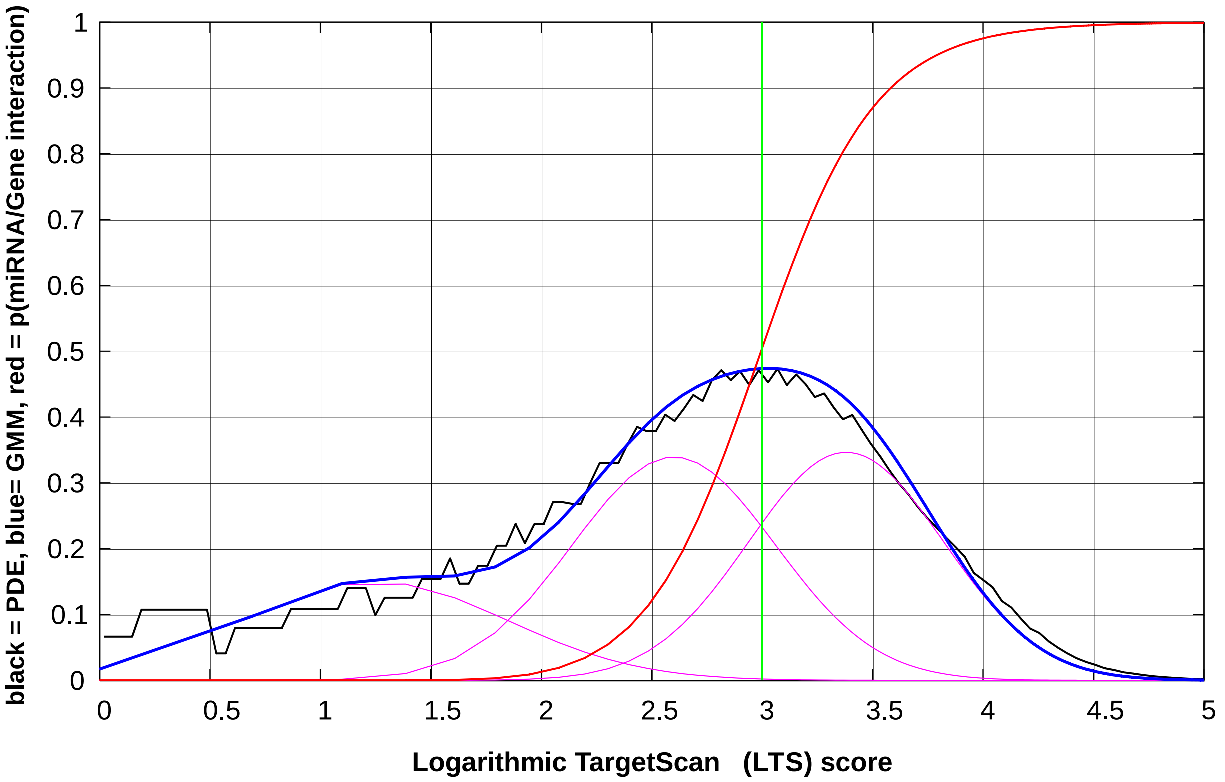

Supplementary Figure 2: Quantile-quantile (Q-Q) plot of the distribution of all TargetScan [1] LTS scores (left panel) and the empirically validated miRNA versus gene interactions right panel). The coincidence with the straight line assures the quality of the model.

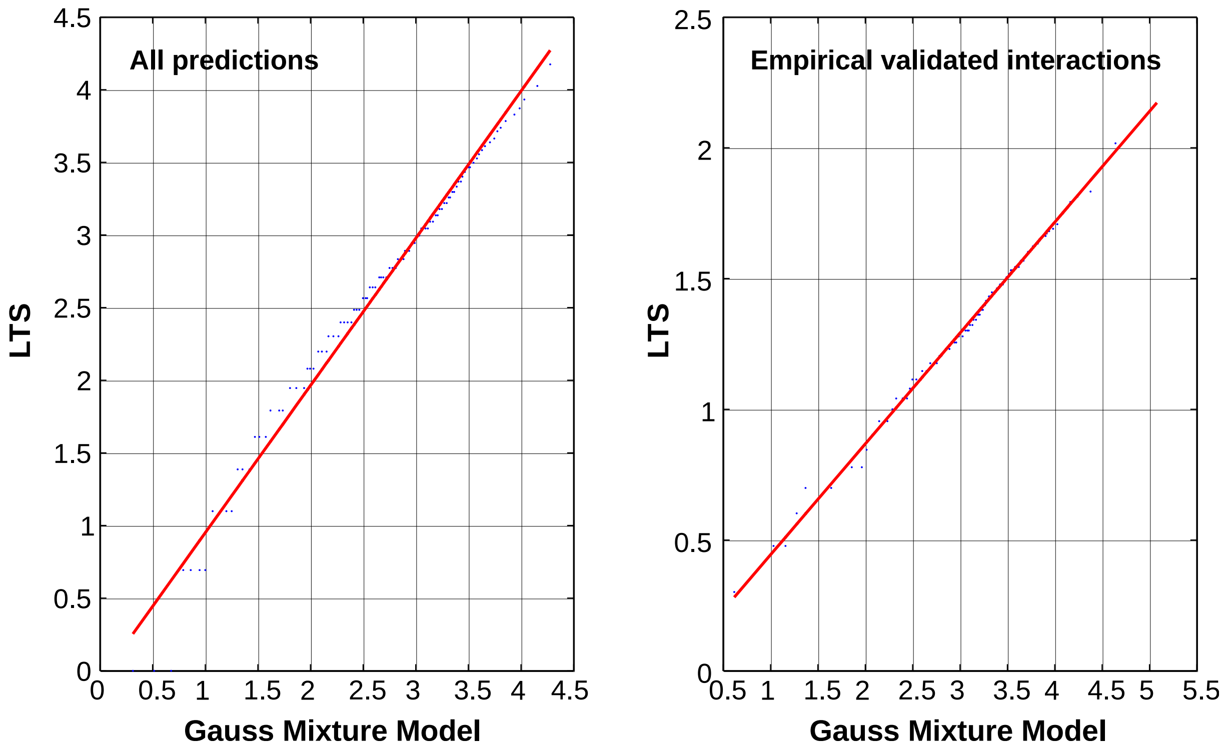

Supplementary Figure 3: Direct comparison of the modeled distributions for TargetScan [1] (LTS) scores (blue) and the empirically validated interactions (green). The vertical line (magenta) indicates a Maximum Likelihood lower limit to acceptable predictions.

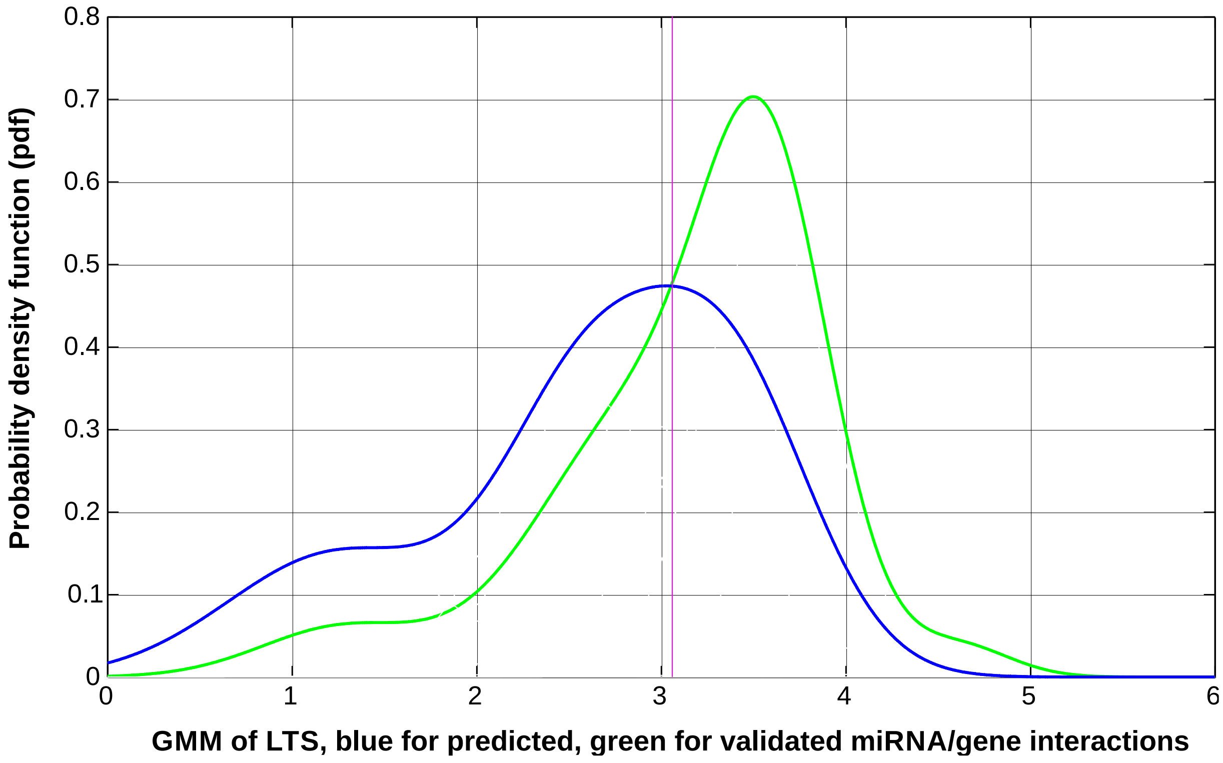

Supplementary Figure 4: Pareto Density Estimation (PDE) of the LTS distribution (blue) together with the p-values (red). PDE is a kernel density estimator particularly suitable for the discovery of mixtures of Gaussians [10]. At $p < 0.02$ (horizontal green dotted line) a plausible limit, $t_{0,}$ for LTS of 4.06 can be assumed.

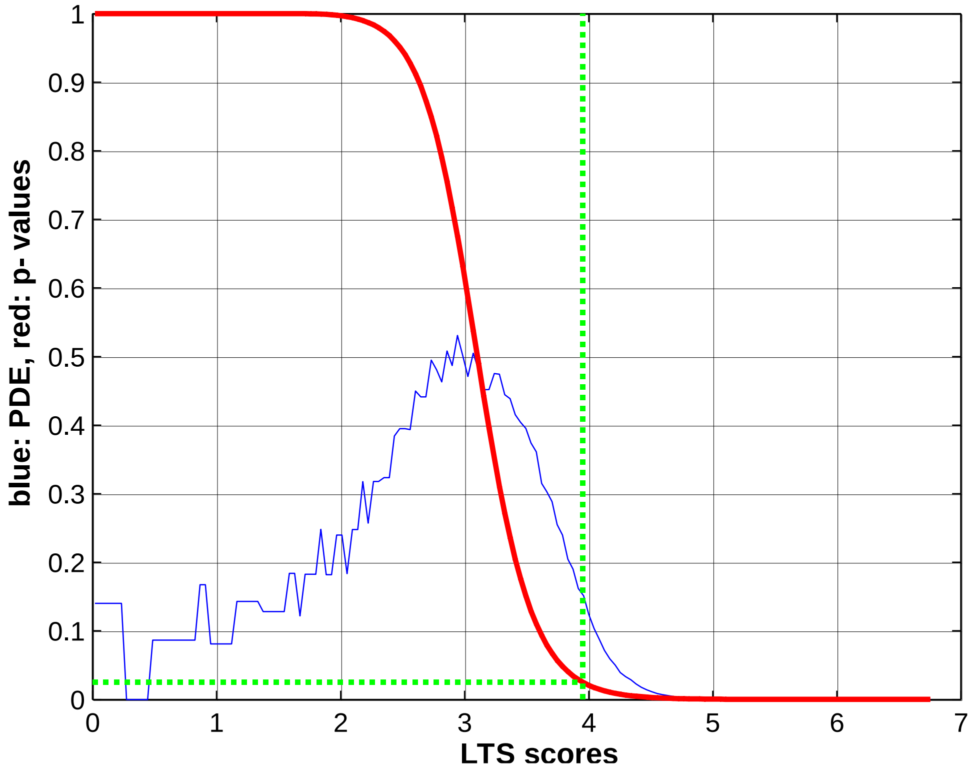

Supplementary Figure 5: The ABC curve [8] of the number of miRNAs per gene. This is a plot of the faction of the largest number of MIRNA per gene interaction versus the fraction of the number of miRNA per genes. The spitting point for sets A and B was determined by the minimum distance from the ABC curve to the point of strongest inequality, i.e. (0,1). This results in set A containing 23 % of all miRNAs (vertical red line) which influence 75 % of all genes (horizontal red line) and set B containing the other 77% of miRNAs which influence only on 25% of the genes.

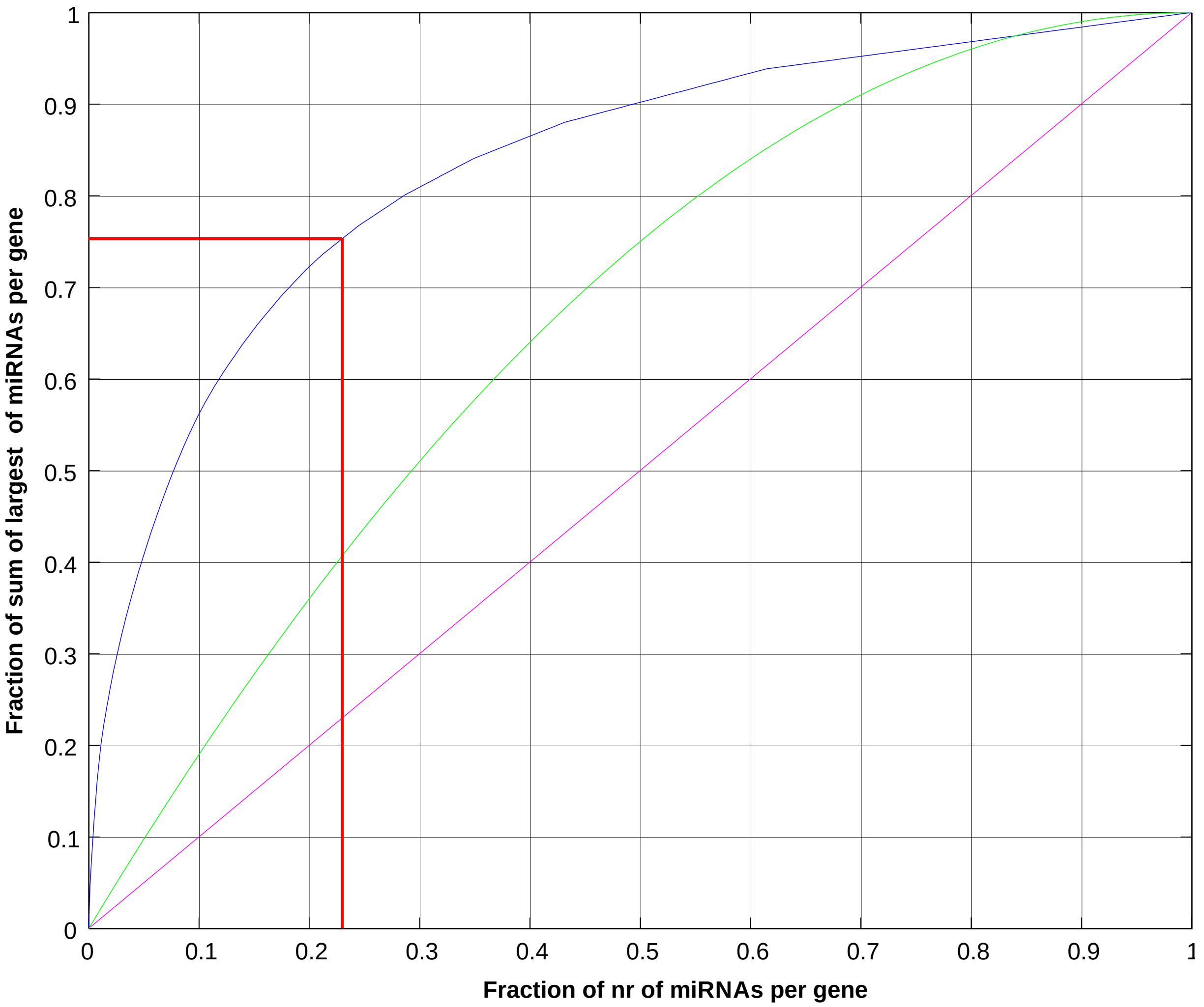

Supplement: Supplementary file 1 — Additional file 1: An appendix with the detailed description of computational prediction of miRNA versus gene interactions. (PDF 798 KB) [file 12864_2014_6856_MOESM1_ESM.pdf]
