# Supplementary figures and images for: What do all the (human) micro-RNAs do?
Source: BMC Genomics. 2014 Nov 18;15(1):976. doi: 10.1186/1471-2164-15-976 (PMC4289375; doi:10.1186/1471-2164-15-976)

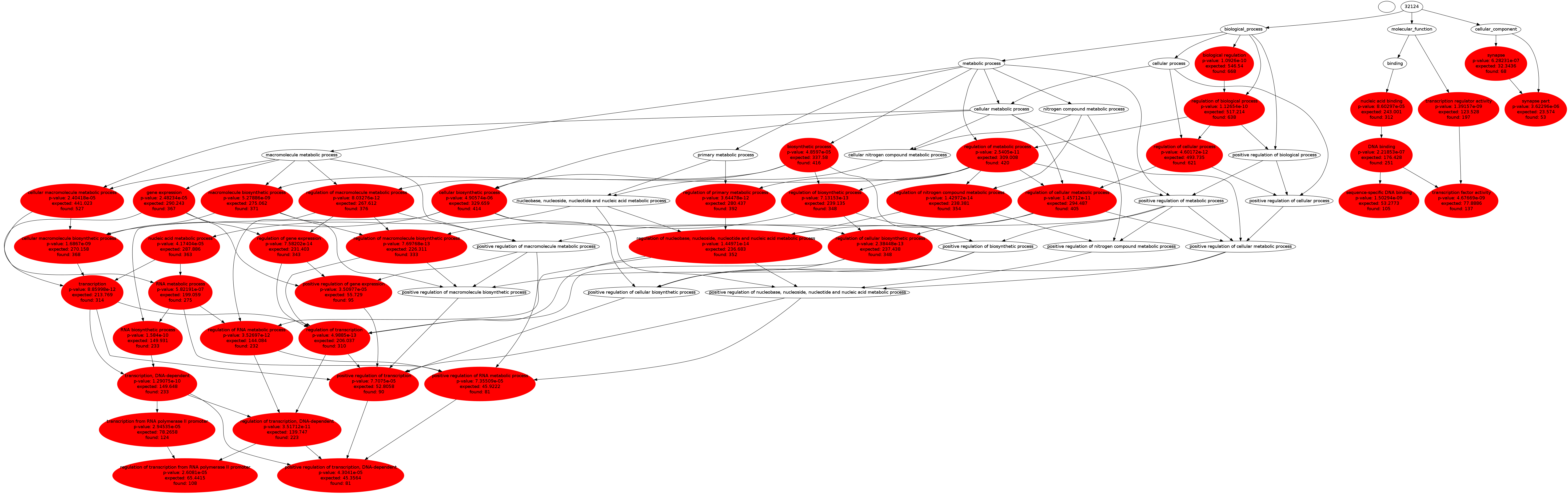

Supplement: Supplementary file 5 — Additional file 5: Figure S3: Displaying the results of overrepresentation analysis of the set of genes with computationally predicted miRNA interaction: ORA_Predicted.png. (PNG 1 MB) [file 12864_2014_6856_MOESM5_ESM.png]
